# Primer Extension Capture: Targeted Sequence Retrieval from Heavily Degraded DNA Sources

**DOI:** 10.3791/1573

**Published:** 2009-09-03

**Authors:** Adrian W. Briggs, Jeffrey M. Good, Richard E. Green, Johannes Krause, Tomislav Maricic, Udo Stenzel, Svante Pääbo

**Affiliations:** Max-Planck Institute for Evolutionary Anthropology, Leipzig

## Abstract

We present a method of targeted DNA sequence retrieval from DNA sources which are heavily degraded and contaminated with microbial DNA, as is typical of ancient bones. The method greatly reduces sample destruction and sequencing demands relative to direct PCR or shotgun sequencing approaches. We used this method to reconstruct the complete mitochondrial DNA (mtDNA) genomes of five Neandertals from across their geographic range. The mtDNA genetic diversity of the late Neandertals was approximately three times lower than that of contemporary modern humans. Together with analyses of mtDNA protein evolution, these data suggest that the long-term effective population size of Neandertals was smaller than that of modern humans and extant great apes.

**Figure:**
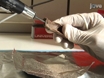


## Protocol

This method was used in the research reported in Briggs *et al., Science* 325 (5938). 318-321 (2009).

### Reagents required:

- AmpliTaq Gold DNA Polymerase
- 10x GeneAmp PCR Buffer II
- MgCl_2_ 25mM
- dNTP mix, 25 mM each
- BSA, 10 mg ml-^1^ in water
- Molecular biology-grade water
- EB buffer (supplied with MinElute PCR Purification kit); 10 mM Tris HCl, pH 8.5
- MinElute PCR Purification Kit
- M-270 streptavidin Dynabeads
- 2x Binding and Wash (BW) buffer (2 M NaCl, 10 mM Tris-Cl, 1 mM EDTA, pH 8.0, 0.2% Tween 20)
- 1x Binding and Wash (BW) buffer (2x BindWash buffer diluted 2X in water)
- Hot Wash (HW) buffer (2.5mM MgCl, 1X Taq Gold buffer, 0.1% Tween 20)

For manufacturer information regarding non-standard reagents and equipment see later table.

### 1) Library amplification

1. The DNA template here is a 454 library prepared from a low copy number DNA source as described in (Rohland and Hofreiter, 2007a, 2007b) and (Maricic and Pääbo, 2009). To amplify the whole library, prepare the following PCR mix:

**Table:** 

**Reagent**	**μl per reaction**
Water	45
10X Gene Amp PCR buffer II	10
MgCl_2_ (25mM)	10
BSA (10 mg / ml)	2
dNTPs (25 mM each)	1
454 emPCR fwd primer/10uM	3
454 emPCR rvs primer/10uM	3
AmpliTaq Gold DNA polymerase (5 U / μl)	1
454 DNA library template	25
**Total**	**100**
2. Use the following PCR program in a Thermo cycler for **14 cycles** amplification
  1. 95°C 12min
  2. 95°C 30s
  3. 60°C (or desired annealing temp) 1 min
  4. 72°C 1 min
  5. Go to 2 (x 13)
  6. 72°C 5min
  7. 10°C ∞
3. Purify the reaction over a Qiagen MinElute spin column according to the manufacturer's instructions. Elute in 50 μl buffer EB.
4. Quantify the purified amplified product as well as an aliquot of the unamplified library with qPCR (Meyer et al., 2007). If the amplified product has more than 1 X 10^12^ copies per ul, reduce the amount of template in the following Primer Extension step to ensure that not more than 2 X 10^12^ copies are added to the Primer Extension reaction.

### 2 ) Primer Extension

1. Prepare a master mix for the required number of reactions.

**Table:** 

**Reagent**	**μl per reaction**
Water	45
10X Gene Amp PCR buffer II	10
MgCl_2_ (25mM)	10
BSA (10 mg / ml)	2
dNTPs (25 mM each)	1
454 emPCR fwd primer/10uM	3
454 emPCR rvs primer/10uM	3
AmpliTaq Gold DNA polymerase (5 U / μl)	1
454 DNA library template	25
**Total**	**100**
2. Run the following program in a Thermocycler for the **single primer extension** reaction:
  1. 95°C 12min
  2. 60°C (or your PEC primer annealing temp if different) 1 min
  3. 72°C 5 min
  4. **72°C forever!CRITICAL STEP** Keep reaction after extension at 72 °C. Then directly pipette 150ul PBI or PB buffer for QIAGEN MinElute purification into the tubes before removing from the heat block. This is important to avoid non-specific primer annealing and capture as the mixture cools.
3. Purify the reaction over a Qiagen MinElute spin column, according to the manufacturer's instructions. Elute in 50 μl EB.

### 3) Extension Product Capture

1. Resuspend stock solution of M-270 beads by vortexing. Take out 25 μl bead suspension per sample. Wash the beads twice with 500ul 2x BW buffer and resuspend in 25 μl 2xBW buffer per sample.
2. Add 25 μl eluate from step **2.3** to 25 μl bead suspension from step **3.1**. Mix then rotate for 15 min at room temperature. *Recommended*: keep remaining 5 ul of the eluate from step **2.3** for qPCR quantification.
3. Transfer the whole mixture to a fresh 1.5ml tube (this helps to reduce carryover of non-target library fragments). Pellet the supernatant using the Magnetic particle collector (MPC) and discard the supernatant.
4. Wash 5 times with 500 μl 1xBW buffer (many washes help to remove as much background as possible). After the last washing step, spin down briefly and remove the last traces of supernatant.
5. Add 500μl 1x Hot Wash (HW) buffer. Shake for 2 min at 65 °C (or 5C above the PEC primer Tm) on a thermal block. Remove the supernatant quickly after this, to minimize cooling down. (This step is to remove background fragments still associated with the PEC primers but not actually extended on during the extension step). Remove the last traces of supernatant.
6. Resuspend the bead pellet in 30 μl EB buffer. Transfer the whole mixture to a fresh 1.5ml tube (this helps to reduce carryover of non-target library fragments). Incubate at 95 °C for 3 minutes in a thermal cycler for elution. Place in the MPC and remove the supernatant, taking care to leave the beads behind.

### 4) Capture Product Amplification

1. Prepare a PCR master mix for the required number of samples.

**Table:** 

**Reagent**	**μl per reaction**
Water	45
10X Gene Amp PCR buffer II	10
MgCl_2_ (25mM)	10
BSA (10 mg / ml)	2
dNTPs (25 mM each)	1
454 emPCR fwd primer/10uM	3
454 emPCR rvs primer/10uM	3
AmpliTaq Gold DNA polymerase (5 U / μl)	1
Eluted capture product	25
**Total**	**100**
2. Use the following PCR program in a Thermo cycler for **14 cycles** amplification:
  1. 95°C 12min
  2. 95°C 30s
  3. 60°C (or desired annealing temp) 1 min
  4. 72°C 1 min
  5. Go to 2 (x 13)
  6. 72°C 5min
  7. 10°C ∞
3. Purify the reaction over a Qiagen MinElute silica spin column according to the manufacturer's instructions. Elute in 50 μl EB buffer. The product can be now used either for a second round of capture, starting at step 2.1, or entered directly into the 454 emulsion PCR protocol, for sequencing.

### Representative Results:

It is strongly recommended when starting out with the PEC protocol to perform first a capture reaction where a small amount of a 'positive control target sequence' (e.g. a ~100bp PCR product - 1 picogram is easily enough) is mixed in with the normal recommended amount of amplified 454 library template (see step **2.1**), and capture is performed with a single PEC primer designed to capture the positive control product. If this control reaction is performed, qPCR with both positive control-specific and 454 adaptor-specific primer pairs can be used to quantify the amount of 'control target' versus background in the purified primer extension reaction (**1.3**), primer extension product (**2.3**) and eluted bead capture product (**3.6**). In this way, both the effectiveness of background removal ("specificity") and the efficiency of target recovery ("sensitivity") in your experimental conditions can be directly measured.

A successful PEC protocol normally has the following properties –

**Sensitivity (**measured by amount of control target retained through the protocol): Total amount of control target in eluted bead capture product (**3.6**) **=~ 1-20%** of total amount in purified primer extension reaction (**2.3**).

**Background** (measured by 454 emPCR primer pair): Total amount of background in eluted bead capture product (**3.6**) **< 0.01%** of total amount in purified primer extension reaction (**2.3**).

If there is less than 1% of the target remaining in the final product, the primer extension step may have been unsuccessful and should be investigated.

If there is much more than 0.01% of the background remaining in the final products, the washing steps may have been incorrectly performed and should be investigated.

## Discussion

The PEC method is simple, quick, sensitive and specific. Therefore we the authors envisage multiple applications outside ancient DNA, such as capture of small RNA fragments from an RNA library, interrogation of structural variation in a pooled sample or capture of 16S (or other loci) diversity from a metagenomic sample. One point to mention is that the sensitivity of capture becomes lower as the number of PEC primers in a multiplex capture reaction increases. PEC is therefore not ideally suited for capture of very large (e.g. a megabase or more) capture regions, but is extremely well suited for capture of small target regions or even SNP positions from many individuals in a rapid fashion.
